# The product of C9orf72, a gene strongly implicated in neurodegeneration, is structurally related to DENN Rab-GEFs

**DOI:** 10.1093/bioinformatics/bts725

**Published:** 2013-01-16

**Authors:** Timothy P. Levine, Rachel D. Daniels, Alberto T. Gatta, Louise H. Wong, Matthew J. Hayes

**Affiliations:** Department of Cell Biology, UCL Institute of Ophthalmology, Bath St, London EC1V 9EL, UK

## Abstract

**Motivation:** Fronto-temporal dementia (FTD) and amyotrophic lateral sclerosis (ALS, also called motor neuron disease, MND) are severe neurodegenerative diseases that show considerable overlap at the clinical and cellular level. The most common single mutation in families with FTD or ALS has recently been mapped to a non-coding repeat expansion in the uncharacterized gene C9ORF72. Although a plausible mechanism for disease is that aberrant C9ORF72 mRNA poisons splicing, it is important to determine the cellular function of C9ORF72, about which nothing is known.

**Results:** Sensitive homology searches showed that C9ORF72 is a full-length distant homologue of proteins related to Differentially Expressed in Normal and Neoplasia (DENN), which is a GDP/GTP exchange factor (GEF) that activates Rab-GTPases. Our results suggest that C9ORF72 is likely to regulate membrane traffic in conjunction with Rab-GTPase switches, and we propose to name the gene and its product DENN-like 72 (DENNL72).

**Supplementary information:** Supplementary data are available at *Bioinformatics* online.

**Contact:** tim.levine@ucl.ac.uk

## 1 INTRODUCTION

Fronto-temporal dementia (FTD) and amyotrophic lateral sclerosis (ALS) are major neurodegenerative diseases that occur in sporadic and familial forms, which show overlap in clinical presentation and cytopathology (Dion *et al.*, 2009). The commonest cause of familial ALS, accounting for 20–40%, maps to a mutation in the gene C9ORF72 at 9p21 (DeJesus-Hernandez *et al.*, 2011; Renton *et al.*, 2011). Mutation of C9ORF72 is also common in patients with familial FTD (10–30%), sporadic FTD (2–10%) and Alzheimer-like dementia (0–4%) (Morris *et al.*, 2012). A single mutation is found: a massively expanded GGGGCC hexanucleotide repeat in an intronic portion of the 5′ untranslated region. In the general population, alleles have a median of two repeats (range: 0–22), which are expanded in pathogenic alleles to >1000 repeats. The pathogenetic mechanism is unknown, but other neurological syndromes arise from non-coding expansions. Pathology could occur via poisoning of splicing factors, in particular TDP-43, with non-coding repeats inducing toxic RNA foci that sequester splicing factors and other nuclear proteins (Todd and Paulson, 2010), as in Fragile X Tremor Ataxia Syndrome (FXTAS) and myotonic dystrophy. TDP-43 deposits and aberrant cytoplasmic mRNA foci in C9ORF72 mutant cells both support a role for splicing (DeJesus-Hernandez *et al.*, 2011; Renton *et al.*, 2011). However, these are not universal findings (Simon-Sanchez *et al.*, 2012), and alternative mechanisms should be considered. One possibility is that the expansion reduces translation, as in Fragile X, consistent with reduced levels of C9ORF72 protein in mutant cells (DeJesus-Hernandez *et al.*, 2011; Renton *et al.*, 2011). Despite its clinical relevance, the normal function of the C9ORF72 protein has remained obscure.

A powerful way to determine protein function is to find homologies to proteins of known function. The sensitivity of the basic local alignment search tool (BLAST) has been increased in several ways. Position-specific iterative (PSI)-BLAST creates a profile of key residues from BLAST, and then searches with increasingly large profiles to converge on a final stable alignment (Altschul *et al.*, 1997). Sensitivity is improved by profile–profile comparison, using profiles for every database entry (Soding, 2005). Because structure diverges far slower than primary sequence (Soding *et al.*, 2005), a further enhancement is to include secondary structural prediction in all profiles. The tool HHpred allocates 15% of the profile weighting at each residue to predicted structure: helix sheet or loop. Importantly, HHpred searches targets not only in the Protein Database (PDB) of solved structures, other databases of domains, but also through proteome-wide libraries of model eukaryotes. Its enhanced profile–profile searches can find homologues that lack significant amino acid conservation even where no structure is solved, and no domain recognized (Hayes *et al.*, 2011).

DENN domain proteins (standing for ‘differentially expressed in normal and neoplastic cells’) are highly conserved Rab-GEFs, with large numbers of homologues in some species: humans have 16 in 7 subgroups (Marat *et al.*, 2011; Yoshimura *et al.*, 2010). Using PSI-BLAST, several DENN-like families have been identified: DENND6 (FAM116), AVL9, Fam45A, LCHN, Afi1p and Anr2p, some of which have GDP/GTP exchange factor (GEF) activity (Linford *et al.*, 2012). All DENN-like proteins contain a domain of 400–500 aa (Marat *et al.*, 2011), the structure of which consists of two lobes that associate intimately with each other and Rab. The tripartite ‘domains’ of u-DENN, c-DENN and d-DENN previously identified *in silico* had no bearing on the structure, and we do not use this nomenclature (Wu *et al.*, 2011). The C-terminal domain makes 16 of 21 Rab contacts, and this lobe is the active GEF. The N-terminal lobe of DENNs adopts a longin domain (LD) ββαβββαα fold, also found in SNAREs, small and medium coat subunits and three types of GEF: three components of the TRAPP complex, both of MON1/CCZ1 and fuzzy (FUZ) (Brooks and Wallingford, 2012; Gerondopoulos *et al.*, 2012; Kinch and Grishin, 2006). LDs, particularly as dimers, are highly conserved platforms for GTPases, deriving from their circularly permuted ancestral roadblock domain, found in prokaryotes and eukaryotes (Miertzschke *et al.*, 2011).

Recently folliculin (FLCN), a tumour suppressor mutated in Birt–Hogg–Dubé syndrome leading to renal cancers, which had no homologies according to PSI-BLAST, was identified as a structural and functional homologue of DENN (Nookala *et al.*, 2012; Wu *et al.*, 2011). Thus, DENN is the founder of a family that is wider than can be appreciated using PSI-BLAST. Here, we show that C9ORF72 is one of several previously unrecognized full-length homologues of DENN proteins, suggesting that C9ORF72 is a member of the DENN-like superfamily, and hence a regulator of membrane traffic.

## 2 METHODS

Online tools used were PSI-BLAST (NCBI), ClustalW2 (EBI), PHYML (T-rex), HHalign, HHpred and CLANS (MPG, Tuebingen). Default settings were used, except in HHpred, which was set to eight iterations, and where query-template alignments were realigned by a Maximum Accuracy algorithm. To optimize HHpred specifically for queries with C9ORF72 and FLCN, parameters were varied for individual searches (see Supplementary Table S2). Protein structure was visualized in QtMG (CCP4).

## 3 RESULTS

### 3.1 Domain structure of C9ORF72

PSI-BLAST seeded with human C9ORF72 converged on 103 sequences including some in protists, indicating that C9ORF72 was present in the last common eukaryotic ancestor (Supplementary Fig. S1A). Insects, plants and most fungi have no C9ORF72, most other species have one, but there are highly expanded sub-families in *Entamoeba* (7) and *Trichomonas* (10). All C9ORF72s have 400–750 aa, and there are no partial homologues. The most conserved residues are distributed throughout the protein (Supplementary Figs S1 and S2). Such conservation in a single block implies that C9ORF72 acts as one functional unit. Because the primary sequence of C9ORF72 shows no homologies, we used six independent structure prediction tools to model C9ORF72 by comparison with the PDB library. Four engines suggested multiple specific homologies, with the only domains that appeared in the results of more than one tool being: M16 peptidases (4), cytochrome bc1 (2), glycohydrolases (2) and DENN (5) (Supplementary Table S1). DENN is represented only twice in the database (*n* = 85, 68 and 476 for the other folds), but was identified by the greatest number of the tools, suggesting a similarity of C9ORF72 to DENN.

### 3.2 C9ORF72 is a full-length homologue of DENN Rab-GEFs

We next used HHpred, which is ideal for detecting remote homology (Soding *et al.*, 2005). This tool aligned the N-terminus of C9ORF72 strongly to LCHN and AVL9, and after optimization of the profile used to generate alignments, a region of 108–229 amino acids near the N-terminus C9ORF72 indicated **p**robability of the **s**ame **s**tructure (p^SS^) = 82–83% (Supplementary Fig. S3), which suggests that C9ORF72 is homologous to the LD in the N-terminus of DENNs.

**Fig. 1.**
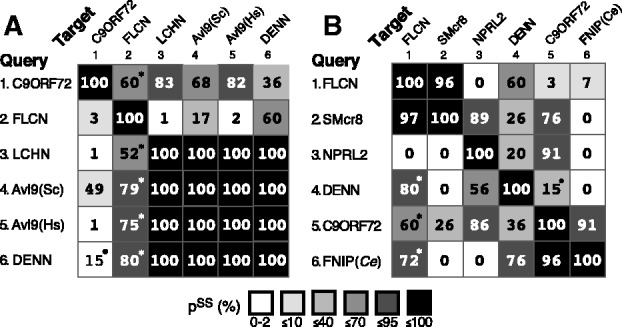
C9ORF72 is structurally homologous to DENN-like proteins. Probabilities of shared structure (p^SS^) of pairwise comparisons of sequences by HHpred, comparing in (**A**): C9ORF72, FLCN, LCHN, two AVL9 sequences (human *Hs*, yeast *Sc*), DENND1B; and in (**B**): FLCN, SMcr8, NPRL2, DENND1B, C9ORF72, FNIP (worm). Values are the maximum p^SS^ obtained after optimizing searches. For details of query alignments and hits, see Supplementary Table S2. Variations: * match is to PDB entry for FLCN;^ •^ match is to worm C9ORF72. Self-searches all give p^SS ^= 100%

Seeding a search with AVL9 (i.e. the reverse search) produced a match that covered the entire length of C9ORF72, although with moderate p^SS^ (49%, Fig. 1A and Supplementary Fig. S3), indicating that the homology might extend to the C-terminus. The C-terminus of C9ORF72 had its strongest matches (p^SS ^> 90%) to FLCN-interacting proteins (FNIPs) 1 and 2, which are regulators of metabolic signalling particularly in B cells (Baba *et al.*, 2006, 2012). The homology between C9ORF72 and FNIPs extends across most of the protein (Supplementary Fig. S3), indicating that C9ORF72 and FNIP are full-length homologues. The homology is strong enough to allow the two profiles to be fully aligned using HHalign, which indicated a significant similarity (E-value = 6 × 10^−^^11^) (Supplementary Table S3). This is significant because FNIP also produces strong hits to DENN and FLCN (p^SS ^= 72 and 76%, Fig. 1B). Thus the indirect relationship via FNIP validates the link between the C-termini of C9ORF72 and DENNs. At the N-terminus, another indirect relationship is found with nitrogen permease regulator-like 2 (NPRL2), a tumour suppressor that regulates Tor (Neklesa and Davis, 2009). NPRL2 aligns strongly to the N-termini of both C9ORF72 and LCHN (p^SS ^= 91 and 95%, Fig. 1B and data not shown), consistent with all three having LDs. Overall, the predicted structural homologues of C9ORF72 are either known DENN homologues, or proteins closely related to them.

### 3.3 C9ORF72 shares full-length secondary structure and primary sequence with DENNs

**Fig. 2.**
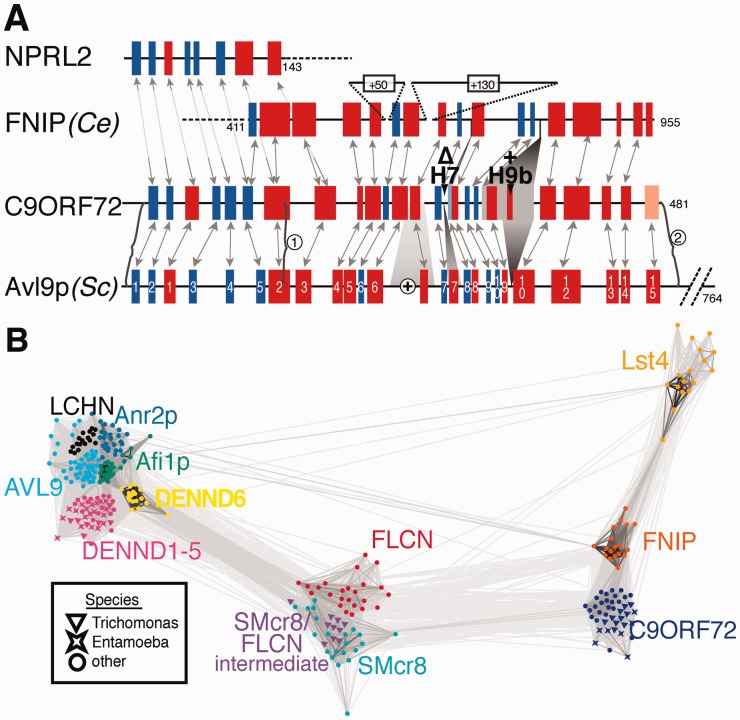
Relationships between C9ORF72, DENNs and other structural homologues. (**A)** Secondary structural conservation between DENN-like proteins and C9ORF72. Predicted helices (red) and sheets (blue, light blue = low confidence) are shown in C9ORF72, Avl9p, NPRL2 N-terminus and FNIP C-terminus. Structural elements are numbered in Avl9p according to homology to DENND1B (Wu *et al.*, 2011). Solid arrows join elements aligned by HHpred, and other alignments were made by hand (dashed arrows). Variations in C9ORF72 are indicated by numbers above. Large brackets indicate the extent of match between C9ORF72 and AVL9 (i) initially and (ii) in the reverse search. Insertions are indicated by the number of residues, except for ‘+’, indicating a loop of 130 aa in human AVL9. (**B)** Cluster map of the DENN-like superfamily. 306 diverse members of 11 sub-families of DENN-like proteins, excluding LD only NPRL2/3s, were tested for all-versus-all similarity (10 iterations of PSI-BLAST), and clustered by CLANS (Frickey and Lupas, 2004). All pairs with similarity threshold *P* ≤ 10^−25^ are joined by lines, which are darker and shorter with greater similarity. Sub-families are identified by colour. Outlier organisms (*Trichomonas* and *Entamoeba*) are identified by shape. Thirty-one colicins included as negative controls showed no links to the other sequences (data not shown)

We next examined the predicted secondary structure of C9ORF72 in detail. Its N-terminus aligns to AVL9 and NPRL2, with an LD fold (ββαβββαα, Fig. 2A). The C-terminus of C9ORF72 contains almost all the secondary structural elements of Avl9p, DENN and FLCN (Fig. 2A). Two features absent in DENND1B are found in both C9ORF72 and FLCN: helix-6b (H6b) before sheet-7 (S7), and lack of H7. The largest feature unique to C9ORF72 is an insertion of a short H9b. C9ORF72 also shows full-length homology to FNIPs, and a novel homologue we found for FLCN, the Smith–Magenis candidate region 8 protein (SMcr8) implicated in autophagy (Behrends *et al.*, 2010) (Supplementary Fig. S4).

A sequence alignment of C9ORF72 with AVL9 and a DENN showed greatest conservation coinciding with predicted structural elements (Supplementary Fig. S5). The binding site of DENNs for Rabs has not been mapped directly, but 21 residues in DENND1B that are likely to be close to Rab have been identified. However, only two contact residues are conserved between DENND1B and other DENN-like proteins (212E and 213R) (Wu *et al.*, 2011). These show partial conservation in C9ORF72 (S/T230 and Q/C231, Supplementary Fig. S2). The most conserved sequence between C9ORF72 and other DENN-like proteins is in two very short motifs that specify tight beta-turns that are likely to be crucial to the overall structure (Supplementary Fig. S6A and S6B). One is a GxxØ motif at S1-S2 that is typical of many LDs (Schlenker *et al.*, 2006), and the other is an Asx motif creating the tight β-turn at S9-S10 (Chou, 2000), which are conserved in C9ORF72s, but not universal in the whole DENN family (Supplementary Fig. S6C).

### 3.4 Relationships between DENN-like proteins

To visualize the relationships between the entire DENN-like superfamily, we created a cluster map using a set of diverse sequences, including fungal and protist. The cluster map showed three major clusters of sequences (Fig. 2B): (i) DENNs together with those DENN-like proteins that are identified by PSI-BLAST; (ii) FLCN and SMcr8; and (iii) C9ORF72, FNIP and Lst4p. Looking at group (i) in more detail, we found that DENND6 is intermediate between other DENNs and FLCN/SMcr8. For group (ii), the finding of sequences with intermediate properties in the protist *Trichomonas* suggests that the FLCN and SMcr8 genes arose by duplication in an early metazoon or pre-metazoal ancestor. For group (iii), it is interesting to note that FNIP has a full-length fungal homologue, Lst4p, E-value = 2 × 10^−^^7^ by HHalign (Supplementary Table S3 and Fig. S4). Note that Lst4p is not related to Lst-4, the sorting nexin SNX9 homologue in *Caenorhabditis elegans*. These overall groupings confirm the strong homologies between new DENN-like proteins found with HHpred, and suggest that C9ORF72 is as divergent from FLCN as FLCN is from DENN.

## 4 DISCUSSION

We have found strong structural homology between C9ORF72 and full length GEF domains in the DENN-like superfamily. At its N-terminus, C9ORF72 is most related to LCHN/AVL9. At its C-terminus, C9ORF72 aligns very well with FNIP, which shows strong homology to DENN and FLCN. Independent of HHpred, multiple independent structural prediction engines suggested DENN as a possible homologue of C9ORF72, despite only two DENN structures being solved to date. As a benchmark to gauge the strength of the matches we obtain, they have higher scores than the matches between DENNs and FLCN. In the future, our prediction can be verified in ways that have been applied to FLCN: solving the structure, and showing GEF activity of purified protein. Our predictions would be helped by including data on the Rab-binding site. However, the lack of conservation of any potential Rab-binding residues between DENND1B and FLCN (Nookala *et al.*, 2012) suggests that it is unlikely that C9ORF72 shares these residues.

Because at least one DENN-like protein (Afi1p in budding yeast) binds an Arf GTPase (Tsai *et al.*, 2008), it is not certain that divergent DENN-like proteins such as C9ORF72 interact with Rabs. However, a clue for the likely targets of C9ORF72 comes from the large families of C9ORF72 in *Trichomonas* and *Entamoeba*. Both these species have highly expanded families of Rabs (Lal *et al.*, 2005; Saito-Nakano *et al.*, 2005), while every other small GTPase family is present in typical numbers (data not shown). This suggests that C9ORF72 interact with Rabs not Arfs.

Fine tuning of membrane trafficking by modulation of Rab activity is already known to be important for neuronal function. Several DENN domain proteins, as well as Als2/Alsin, a Rab5-GEF from a different protein family, have been linked to neurodegeneration because of their effects on membrane trafficking (Azzedine *et al.*, 2003; Del Villar and Miller, 2004; Hadano *et al.*, 2010). Endosomal and lyosomal function are increasingly recognized as sites of cellular dysfunction in ALS/FTD because of their role in two specific aspects of membrane traffic. One is autophagocytosis, which is critical for pathways that clear large protein aggregates, and which is required for many aspects of neuronal survival (Harris and Rubinsztein, 2012). Another is endocytic trafficking of the growth factor progranulin (PGRN), uptake of which by some cells might lead to defective neurotrophic support for key neurons (Carrasquillo *et al.*, 2010). TDP-43 function is linked to this process via splicing of sortilin (SORT1), the PGRN uptake receptor (Prudencio *et al.*, 2012), so a role for C9ORF72 in sortilin-positive endosomes should now be considered. Furthermore, Lst4p, the closest relative of C9ORF72 in yeast, works in the same pathway as Lst7p, yeast FLCN, to prevent vacuolar (lysosomal) delivery and re-direct cargo from endosomes back to the trans Golgi network and plasma membrane. If C9ORF72 shares functions with Lst4p, human cells with reduced C9ORF72 may well have excess routing of sortilin/PGRN to lysosomes.

Given the strong homology we have found, we suggest that C9ORF72 should be renamed DENN-like 72 (DENNL72). Future studies into DENNL72 should investigate which Rabs it interacts with, and demonstrate its GEF activity, as well as focussing on membrane traffic in cells both in patients with the pathogenic mutation, and in model organisms, including yeast lacking Lst4p.

## Supplementary Material

Supplementary Data
