# Supplementary material for: The product of C9orf72, a gene strongly implicated in neurodegeneration, is structurally related to DENN Rab-GEFs
Source: Bioinformatics. 2013 Jan 16;29(4):499–503. doi: 10.1093/bioinformatics/bts725 (PMC3570213; doi:10.1093/bioinformatics/bts725)
Supplement: Supplementary Data [file supp_29_4_499__index.html]

The product of C9orf72, a gene strongly implicated in neurodegeneration, is structurally related to DENN Rab-GEFs — The product of C9orf72, a gene strongly implicated in neurodegeneration, is structurally related to DENN Rab-GEFs — Supplementary Data 

# The product of C9orf72, a gene strongly implicated in neurodegeneration, is structurally related to DENN Rab-GEFs

## Supplementary Data

files

**Files in this Data Supplement:**

- Supplementary Data - doc file
