# Supplementary material for: The product of C9orf72, a gene strongly implicated in neurodegeneration, is structurally related to DENN Rab-GEFs
Source: Bioinformatics. 2013 Jan 16;29(4):499–503. doi: 10.1093/bioinformatics/bts725 (PMC3570213; doi:10.1093/bioinformatics/bts725)
Supplement: Supplementary Data [file supp_bts725_Supplementary_Information_Levine_et_al.doc]

**SUPPLEMENTARY INFORMATION**

for

“The product of C9orf72, a gene strongly implicated in neurodegeneration, is structurally related to DENN Rab-GEFs”

(Levine  *et al.*)


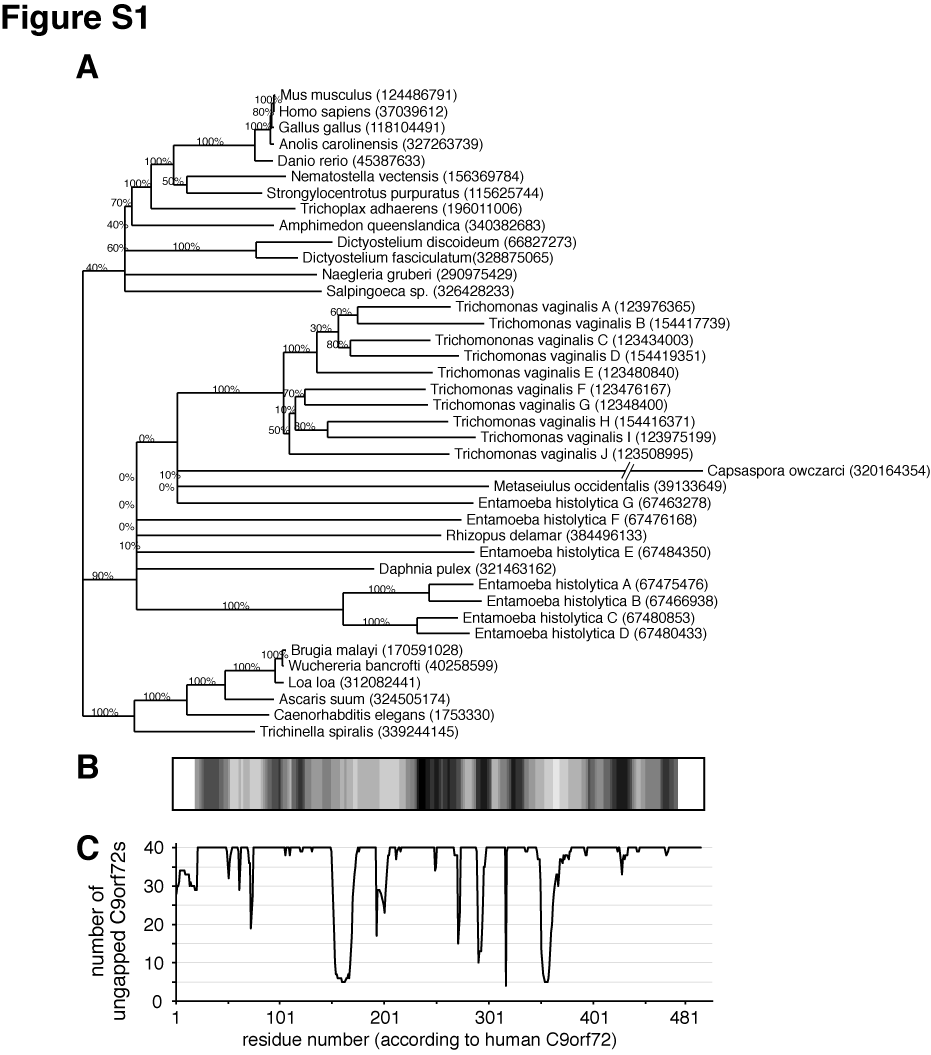
**Supplementary Figure 1: C9orf72 is a conserved protein that functions as a single unit.**

­

**A. Phylogenetic tree of the C9orf72 family.**

103 sequences were obtained by PSI-BLAST seeded with human C9orf72 after 8 iterations. Sample sequences from each phylum were chosen, except for *E. histolytica* and *Trichomonas* where all sequences were included, and the resulting 40 sequences were aligned at T-Rex by PHYML . Numbers indicate bootstrap values. The mite (*Metaseiulus*), fungal (*Rhizopus*), *Daphnia* and *Capsaspora* (branch shortened) sequences are found among the *Entamoeba* sequences, but with zero bootstrap values, indicating that they are not specifically related to any other sequence. However, all of these sequences are true positives, in that they produce BLAST hits only to the other members of this family. *E. dispar* and *E. nuttalli* code for sequences closely paired with all those in *E. histolytica* (data not shown). Multiple ancient duplication events are evident in *Entamoeba*, and to a lesser extent in *Trichomonas*. A full alignment is shown in Supplementary Figure 2.

**B. Conservation in C9orf72 sequences**

Aligned sequences from (A) were assessed for conservation of physico-chemical properties of amino acids according to the AMAS method and a local average ±4 was plotted.

**C. Regions of C9orf72 with sequence conservation are ungapped**

For each aligned position the number of sequences contributing a residue, as opposed to creating a gap, was determined. The two regions, identified by asterisks, correspond to the regions in Figure S5 where the vertebrate sequence contains inserts.

**Supplementary Figure 2: Alignment of diverse C9orf72 homologues**

**A. Residues 1-297**

**
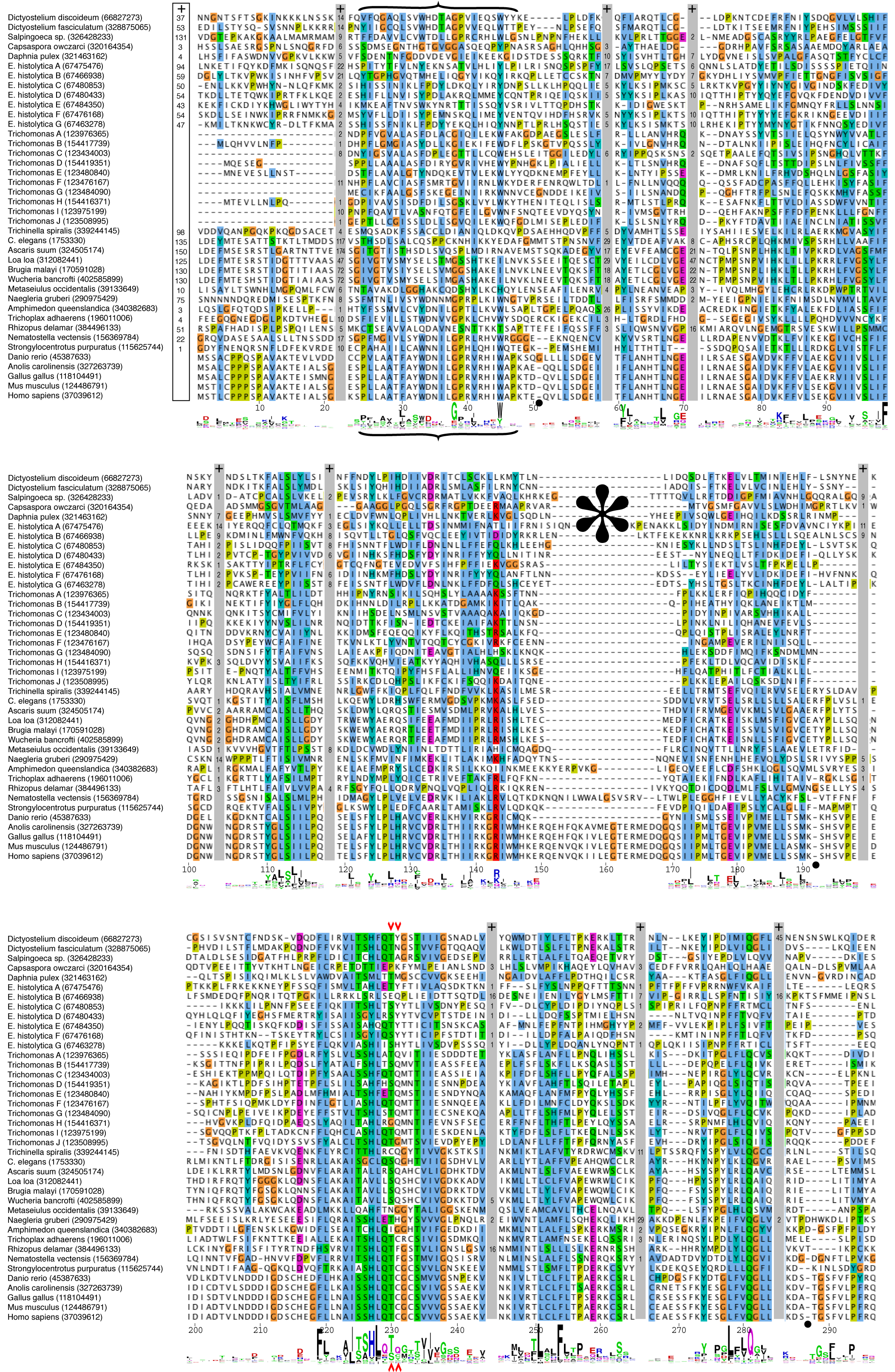
**

**Supplementary Figure 2B: Residues 298-481**

40 diverse C9orf72 sequences (see Supplementary Figure 1) were aligned by ClustalW2. Both flanks were excised (white rectangles), as were all 11 inserts that had at least 10 unaligned residues in one sequence (grey rectangles, numbers indicate the excised residues). Five positions indicated by “•” are where single insertions have been made in human C9orf72. At the bottom is a consensus logo created at weblogo.berkeley.edu. Asterisks indicate two regions present only in vertebrates. Numbering indicates the position in the human sequence. Brackets show residues aligned in Supplementary Figure 3. Red arrowheads indicate the equivalent to E–R (212-3) in DENND1B. Both the *Rhizopus* and *Wucheria* genomes have been annotated as dividing C9orf72 sequences into two adjacent genes, which are shown here as single gene products. In addition to the single *Rhizopus* fungal C9orf72, there is another in the un-annotated genome of the zygomycete *Phycomyces*.

**Supplementary Figure 3: Predicted structural homologues of C9ORF72**

­­
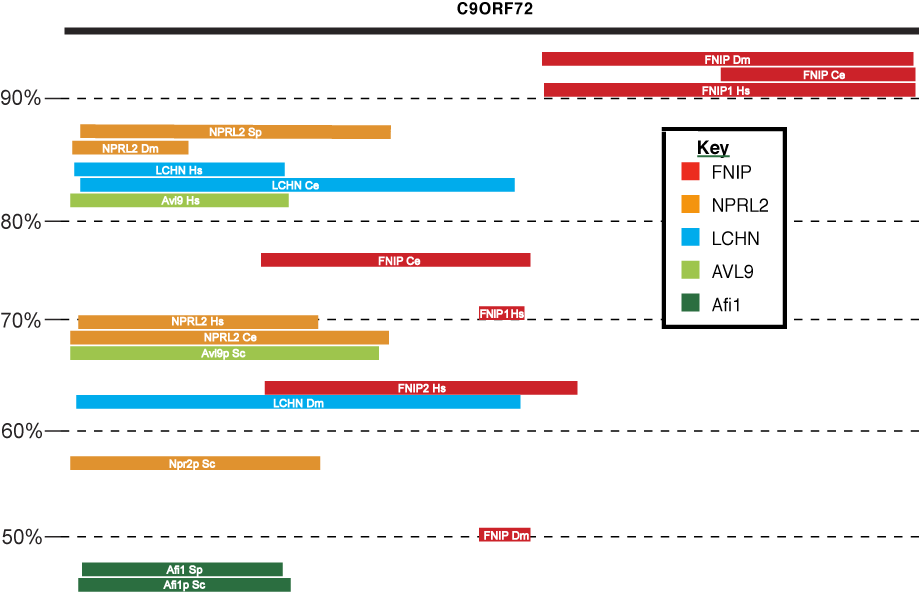


The results of a single HHpred search are shown. The query was C9ORF72 from worm (T04C4.1b, 734 aa), from which two inserts (1-28 & 67-318) were removed. The alignment obtained from HHblits (8 iterations) contained 34 sequences, from which 5 highly repeated (vertebrate) sequences were deleted. The remaining 29 aligned sequences (see Supplementary Table 3) were used to search targets of human, fly, worm, budding and fission yeast (*Hs*, *Dm*, *Ce*, *Sc*, *Sp*). Independent hits with p^SS^>40% are shown along a vertically oriented scale of increasing p^SS^, coloured by protein family: FNIP, LCHN, AVL9, NPRL2 and Afi1.

By comparison, in a non-optimised query with human C9ORF72 (all residues), the results obtained are similar in overall pattern, but have lower p^SS^ values, with the top DENN hit being Avl9p (p^SS^=74%; 109 columns) and the top hit overall being worm FNIP (p^SS^=92%; 93 columns).

| **Systematic name** | **Species** | **Protein** | **UniProt code** | **GI number** |
| --- | --- | --- | --- | --- |
| T04C4.1 isoform b | C.elegans | FNIPb Ce | Q95QK5 | 351064675 |
| LOC96459 isoform 1 | H.sapiens | FNIP1 Hs | Q9P278 | 57164964 |
| LOC96459 isoform 2 | H.sapiens | FNIP1 Hs | Q9P278 | 57164966 |
| CG3764 | D.melanogaster | FNIP Dm | Q9VVC7 | 24665700 |
| T04C4.1 isoform a | C.elegans | FNIPa Ce | H2L0I8 | 86574953 |
| similar to Nitrogen Permease Regulator | S.pombe | NPRL2 Sp | O42857 | 19114705 |
| CG9104 | D.melanogaster | NPRL2 Dm | Q9VXA0 | 18859975 |
| LCHN | H.sapiens | LCHN Hs | A4D1U4 | 122937185 |
| T26A5.6 | C.elegans | LCHN Ce | Q22794 | 25148986 |
| AVL9 homolog | H.sapiens | Avl9 Hs | Q8NBF6 | 57529131 |
| tumor suppressor candidate 4 (NPRL2) | H.sapiens | NPRL2 Hs | Q8WTW4 | 50592992 |
| F49E8.1 | C.elegans | NPRL2 Ce | Q20633 | 71989058 |
| Avl9p | S.cerevisiae | Avl9 Sc | Q12500 | 6323143 |
| CG11178 isoform a | D.melanogaster | LCHN Dm | Q9VYB2 | 24641817 |
| Npr2p | S.cerevisiae | NPR2p Sc | C7GXE9 | 37362640 |
| similar to Sc Ykl047w | S.pombe | Afi1 Sp | O42907 | 19112090 |
| Ykl047wp | S.cerevisiae | Afi1p Sc | G2WHY5 | 6322804 |

**Supplementary Figure 4: Secondary structural predictions for newly described homologues of Rab-GEFs**

LD


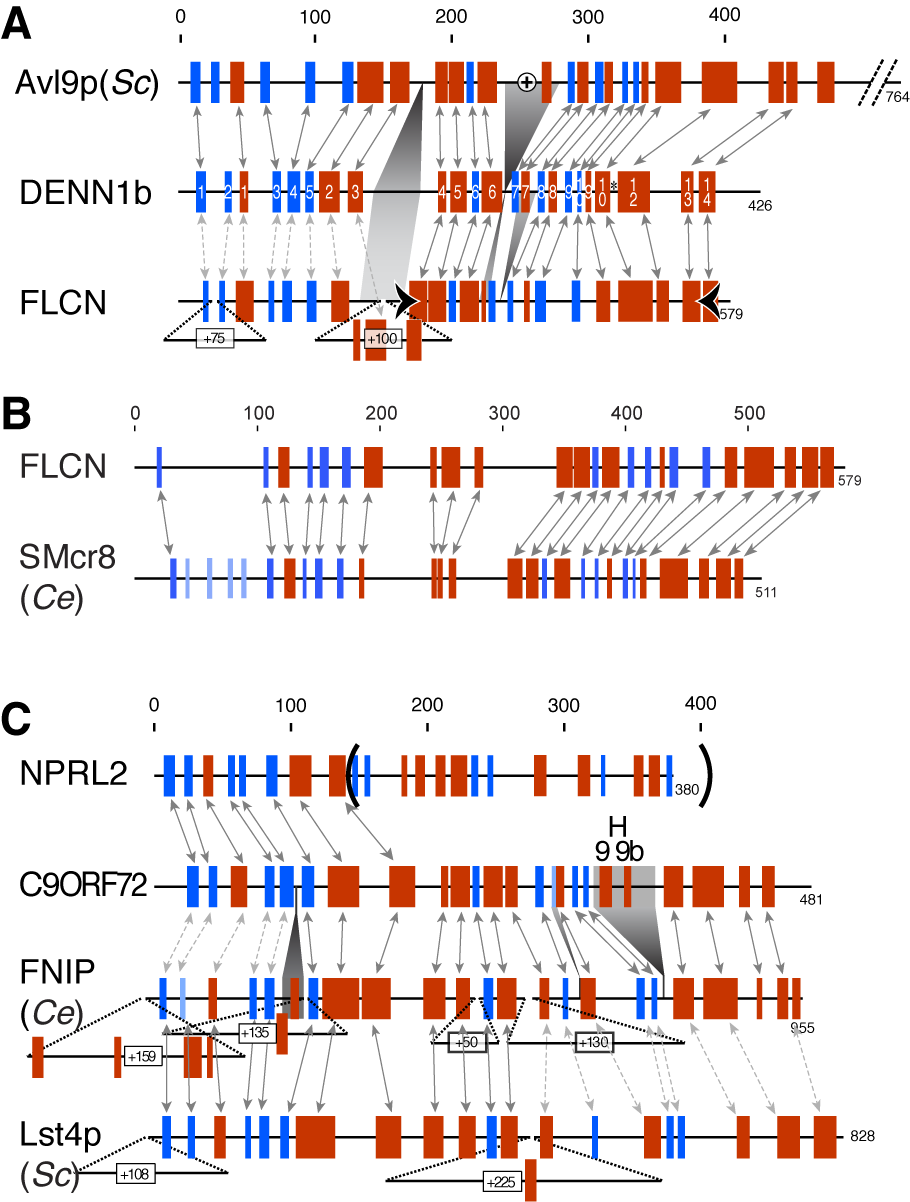


DENN-like proteins and C9ORF72 homologues were aligned as in Figure 2 according to predicted secondary structural elements. **(A)** Known DENNs: FLCN, DENND1B and Avl9p. The DENN1B solved structure contains 10 beta sheets and 14 helices (numbered), however H11, which is a short (3 residue) 3-10 helix between H10 and H12, is not identified by HHpred and has been omitted. The solved portion of FLCN (3v42, residues 341-567) is indicated by arrowheads. Regions that do not align are shown as looped inserts. **(B)** FLCN and SMCr8. Here FMCN is shown without gaps to indicate the similarity of secondary structural elements with SMcr8. **(C)** C9ORF72, NPRL2, FNIP and Lst4p. Unlike in Figure 2, here the whole of NPRL2 and two FNIPs (in *C. elegans*, T04C4.1b; in yeast Lst4p) are shown, with unaligned inserts looped out. Brackets in NPRL2 indicate the portion that does not align. Two helices in C9ORF72 are absent from FNIP and Lst4p. Proteins more closely related to DENN than AVL9 (LCHN, FAM116, FAM45, Anr2p, Afi1p) are not shown as they all share every structural element of DENND1B, except LCHN lacks H14. Although the N-terminus of FLCN has not been crystalised, the structural elements to form an LD are detectable, with insertions at S1-S2 and H2-H3. The extent of the LD is demonstrated by the black bar above.

**Supplementary Figure 5: Sequence alignment of C9ORF72 with Avl9p and a DENN**

**
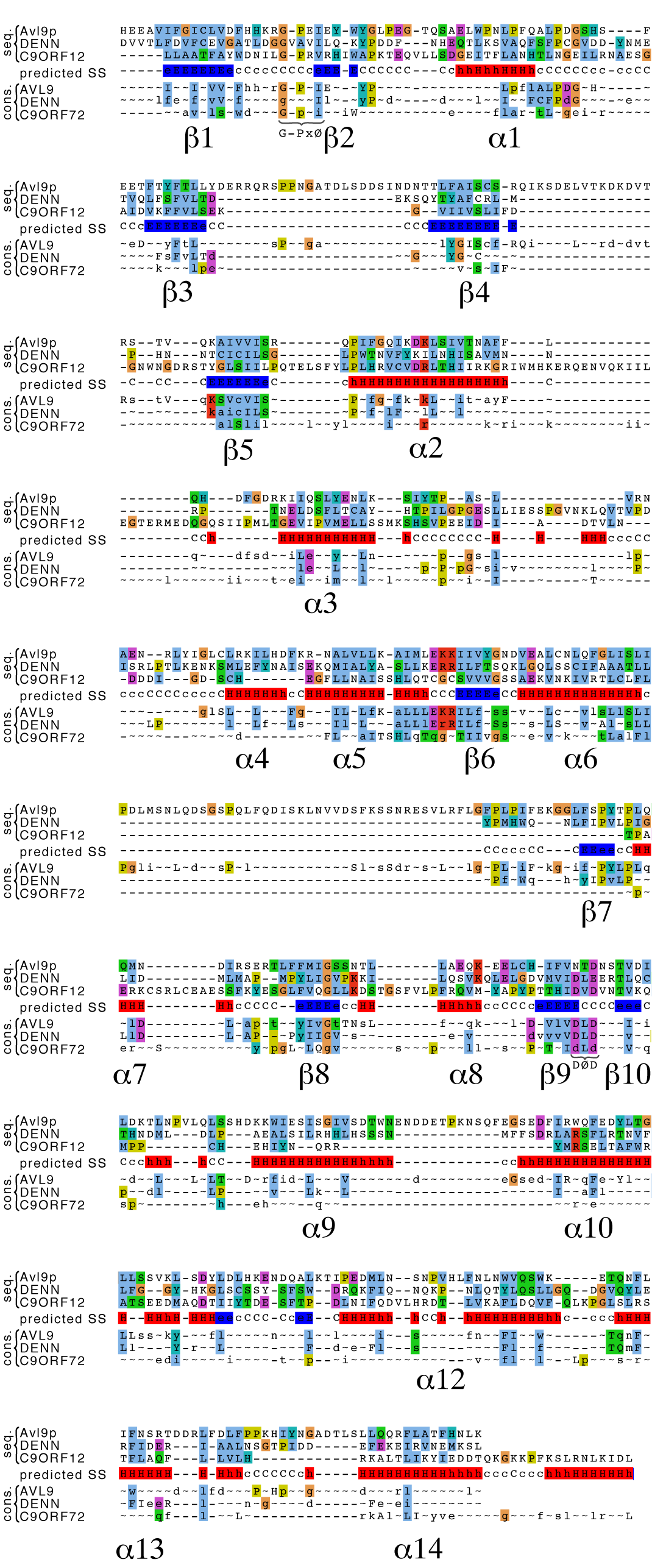
**

Residues 25-435 of C9ORF72 were aligned with Avl9p and a representative DENN domain by merging separate alignments of Avl9p to C9ORF72 and DENND1A (*Brugia malayi*) to C9ORF72. Also shown is the predicted secondary structure, and the consensus for each sequence, *i.e.* the most highly conserved residues for the three profiles. Weaker predictions are in lower case. Residues within each trio of sequences or consensuses are coloured by conservation according to the Clustalx scheme. C9ORF72 has conserved residues throughout, particularly in or near predicted secondary structural elements. Avl9 and the DENN show greater similarity to each other.

**Supplementary Figure 6: Structural motifs conserved in the DENN-like superfamily**


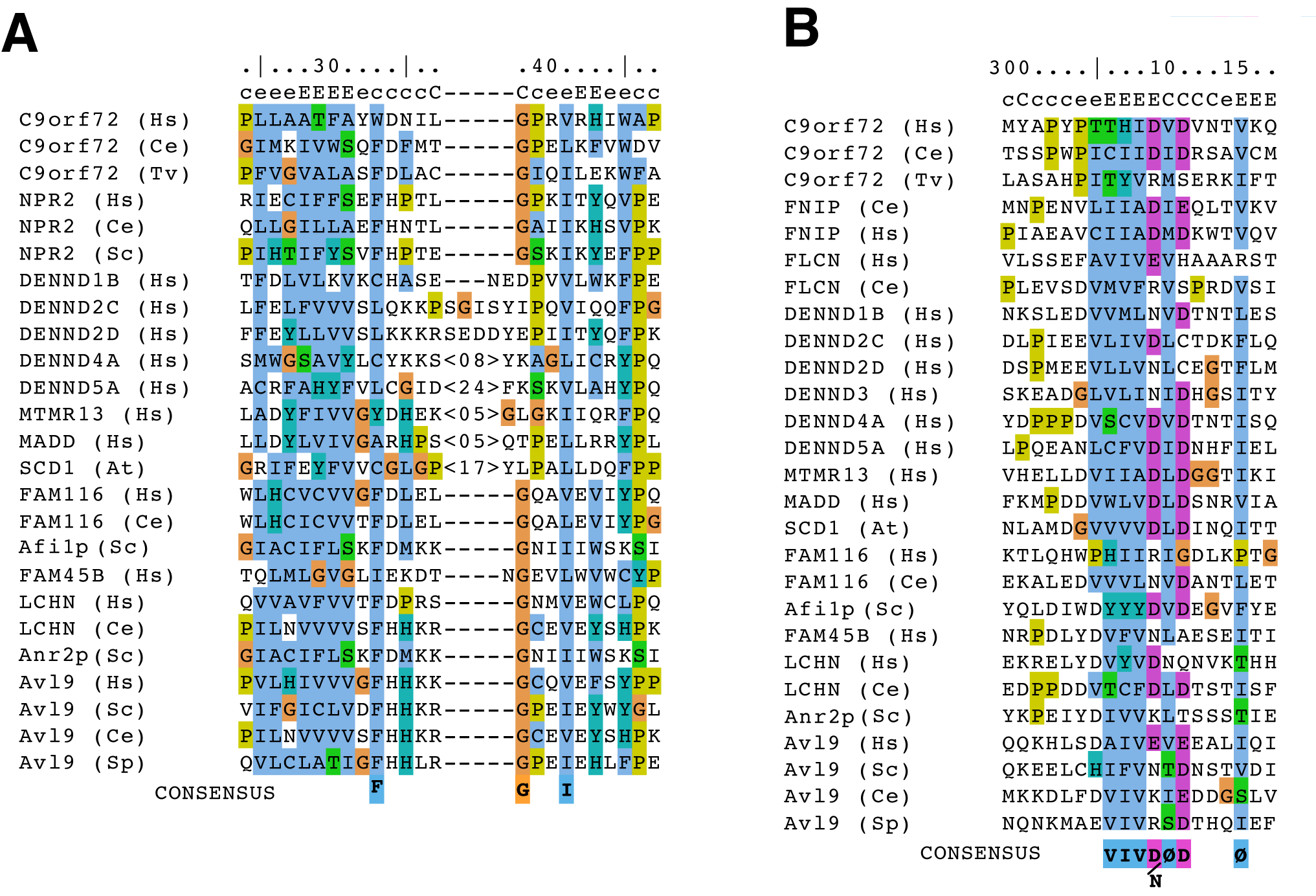
­

­

**A and B.** Alignments of conserved sequence motifs (A) S1–S2, (B) S9–S10. Sequences from DENNs, DENN-like proteins, C9ORF72 NPRL2 (A only) and FNIP (B only) were aligned, coloured according to the Clustalx scheme, and used to calculate a consensus (bottom). Ø indicates a conserved hydrophobic. Structure and numbering according to C9ORF72 is shown at the top. In (A), DENND4 and FLCN sequences were omitted because it wa­s not possible to assign S1. In (B),” **•**” indicates an insertion (for example 24 residues have been omitted for FLCN). Species: Hs – human, Ce – worm, Sc – budding yeast, Sp – fission yeast, Tv – *Trichomonas*, At – *Arabidopsis thaliana*.


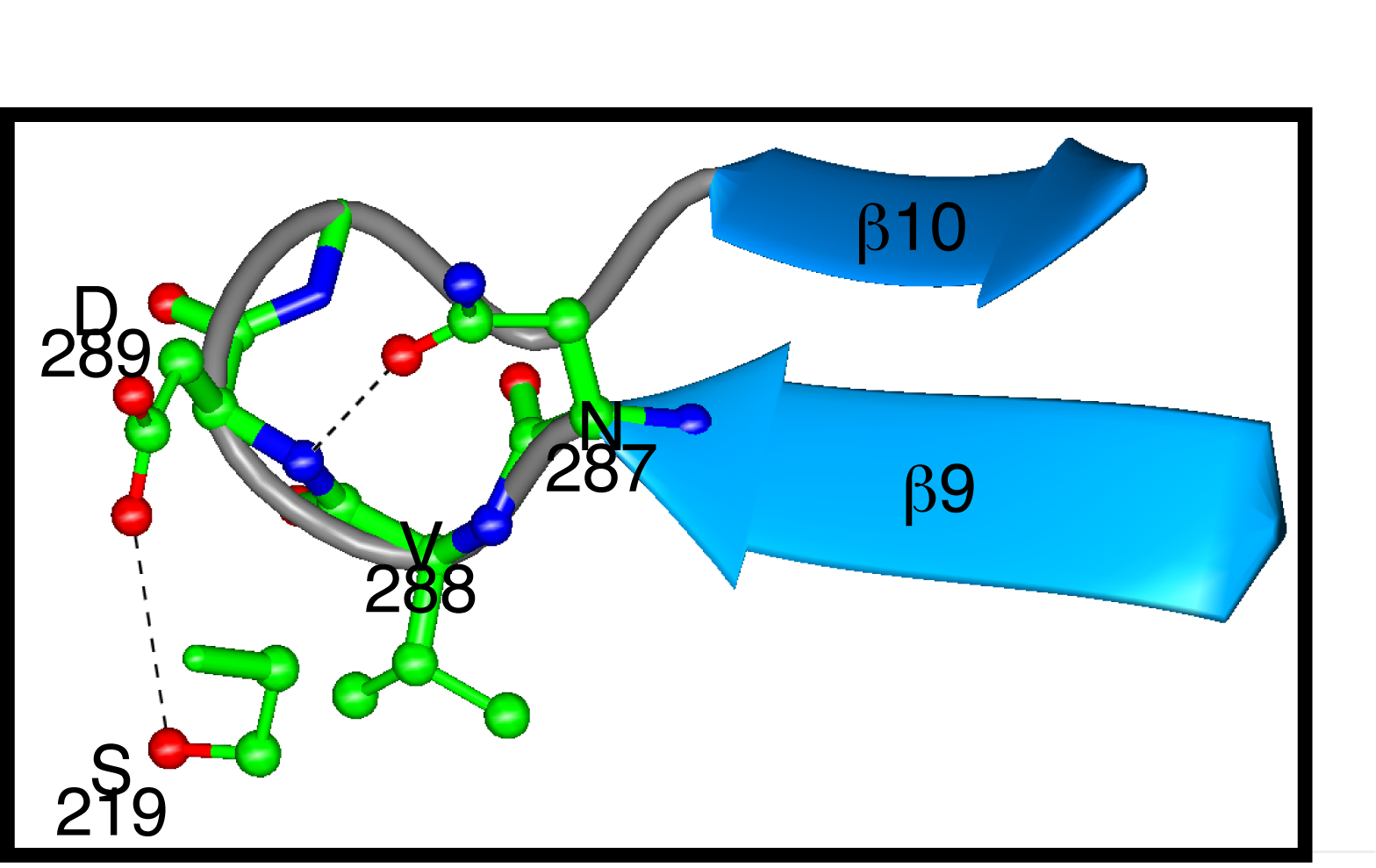


**C**

**C.** An Asx motif in DENN1B facilitates a tight turn between S9 and S10. The diagram shows amino acids 287-289 (N–V–D, see black box in B) and adjacent molecules in DENND1B . The Asx motif creates an 9 atom ring that is completed by a hydrogen bond between the hydroxyl of the asparagine-287 side-chain and the backbone nitrogen of aspartate-289. The side chain of aspartate-289 is stabilised by a hydrogen bond to serine-219. Asx motifs allow either aspartate or asparagine at position 1 . C9ORF72 can form the same tight turn using D–V–D (309-311, see red box in B) and serine-239.

**Supplementary Table 1: Modelling C9orf72 on solved structures**

| Raptor | SAM-T08 | Galaxy * | I-TASSER (Human):  analogues | I-TASSER (Human): templates | I-TASSER (Worm):  analogues |
| --- | --- | --- | --- | --- | --- |
| 1ezvA – cyt | 3c1dA – R | 3tw8A – D | 1q2lA – P | 1q2lA – P | 3tw8A – D |
| 3gwb – P | 3dfgA – R | 1pp9B – P | 2g49B – P | 2fgeA – P | 1tr2B – Vin |
| 1sqbB – P | 3tw8A – D | 3amjB – P | 2g56B2 – P | 1hr6A – P | 1st6A – Vin |
| 1hr6B – P | 3b2cA – C |  | 3s5hA – P | 3tw8A – D | 1llwA – gltS |
| 3cwbA – P | 2ns9A – S |  | 2fgeA – P | 1ezvA – cyt | 3cv5A – gltS |
| 3eoqA – P | 3h3lA – G16 |  | 1hr7A – P | 1gw5B – A | 3tonA – G31 |
| 1hr7B – P | 2nutB – W |  | 1hr9F – P | 1hr6B – P | 3lppA – G31 |
| 1bccB – P | 2pcsA – S |  | 3amjC – P | 3m1iC – E | 1bxrA – CPS |
| 3tw8A – D | 3b0sA – C |  | 3cwbA – P | 2i8bA – V | 1t3tA – purM |
| 3cwbB – P | 1f6yA – Pt |  | 1l0nA – P | 1a4yA – L | 3lvtA – CE4 |

The table shows the lists of top matches returned after submission of human C9orf72 to Raptor , SAM-T08 , Galaxy (* no order specified) , and, I-TASSER , which returned two lists, one of analogues and one of templates that match the query. The final column shows the I-TASSER analogues for the worm C9orf72 homologue F18A1.6a. None of the significance values were highly significant (not shown).

The functions of the domains identified in each case (together with the total number of domains in PDB with this fold) are: A = Adaptor Protein AP2 complex subunit beta-1 (17); C = Collagen-like (93); CE4 = carbohydrate esterase 4 superfamily (103); CPS = Carbamoyl-phosphate synthetase (162); cyt = cytochrome BC1 (68); D = DENN (2); E = Exportin-1 CRM1 C-terminal (26); G16 = glyco-hydrolase type 16 (50); G31 = glyco-hydrolase type 31 (426); GltS = glutamate synthase (35); L = Leucine Rich Repeats (199); P = peptidase M16 (85); Pt = Pterin binding enzyme (94); purM = PurM-like Superfamily (41); R = RecX (4); S = StART superfamily (220); V = Ebola virus-specific transcription factor VP30 (2); Vin = vinculin superfamily (84); W = von Willebrand factor type A domain (284).

Two other tools were used: (A) QUARK returns 10 models for a query of maximum length 200 aa without listing similar structures. We submitted residues 23-222, which were modelled as various alpha-beta forms; (B) Phyre2 was also used, but the results were not helpful, only finding short homologies below 30 aa in length.

The only four folds that appear in separate columns (with number of columns) are: peptidase M16 (4), cytochrome BC1 (2), glyco-hydrolases (2) and DENN (5).

All candidates that occurred multiple times were tested in HHpred, and none showed any match to C9ORF72 (*i.e.* p^SS^<1%).

**Supplementary Table 2: Details of searches between C9ORF72 and DENN-like proteins**

**A**

|  | TARGET | | |
| --- | --- | --- | --- |
|  | C9orf72 | FLCN (3v42) | LCHN |
| QUERY ⇓ | pSS N <query><target> | pSS N <query><target> | pSS N <query><target> |
| C9orf72 |  | 60 178 193-398 22-210 | 83 85 5-112 72-160 |
| FLCN | 3 53 364-416 224-277 |  | 1 48 346-394 232-280 |
| LCHN | 1 61 387-447 399-461 | 52 48 232-280 6- 54 |  |
| Avl9 (Sc) | 49 374 1-485 1-481 | 79 55 127-181 7- 62 | 100 274 3-359 71-365 |
| Avl9 (Hs) | 1 38 196-237 226-263 | 75 49 178-226 7- 56 | 100 267 12-477 73-361 |
| DENN | 15 79 207-295 503-588 | 80 97 188-291 7-104 | 100 254 7-300 71-347 |
|  |  |  |  |
|  | TARGET | | |
|  | Avl9p (Sc) | AVL9 (Hs) | DENN |
| QUERY ⇓ | pSS N <query><target> | pSS N <query><target> | pSS N <query><target> |
| C9orf72 | 68 130 3-160 2-169 | 82 89 3-114 9-101 | 36 222 37-289 451-719 |
| FLCN | 17 56 348-404 187-242 | 2 53 348-401 179-231 | 60 71 347-424 169-252 |
| LCHN | 100 349 72-446 4-450 | 100 305 73-406 12-522 | 100 428 3-452 324-867 |
| Avl9 (Sc) |  | 100 441 5-491 12-589 | 100 341 2-454 6-371 |
| Avl9 (Hs) | 100 442 11-589 4-491 |  | 100 212 1-244 1-232 |
| DENN | 100 337 7-386 3-452 | 100 343 7-417 10-571 |  |

Each square shows the p^SS^ (also given in Figure 1A) number of columns matched (N), extent of match both in query (<query>) and in target (<target>). The long diagonal of self-searches is omitted (all p^SS^=100%). Unless otherwise stated HHpred was seeded with the full-length query and 8 iterations of HHblits were used with MAC realignment. All the strongest matches for FLCN were to the solved structure 3v42 . For DENN, DENND1B was the query The top hits to members of the DENN family were: C9orf72🡪DENND2C, FLCN🡪3tw8 (DENND1B), LCHN🡪DENND4B, Avl9 (both)🡪DENND1A.

Specific variations: for C9orf72🡪FLCN and DENN the query list contained the 40 sequences in Supplementary Figure 2, missing the human and mouse; for C9orf72🡪LCHN and AVL9s the query list was based on T04C4.1b (*C.elegans*) with inserts removed (leaving 435 aa), which identified 34 sequences in HHblits, 5 of the 8 vertebrate sequences were then omitted, leaving 29 sequences (see Supplementary Table 4); for all searches with FLCN, the query set was curated to remove 3 outliers; for LCHN🡪C9orf72, a stronger match was obtained with 2 iterations; for Avl9p🡪FLCN only residues 60-359 of Avl9 were included in the query; for Avl9p🡪C9ORF72 reducndant and short sequences were removed from the query alignment; for DENN1B🡪C9orf72 the match is to worm C9orf72 (F18A1.6a);

**2B**

|  | TARGET | | |
| --- | --- | --- | --- |
|  | FLCN | SMcr8 | NPRL2 |
| QUERY ⇓ | pSS N <query> <target> | pSS N <query><target> | pSS N <query><target> |
| FLCN |  | 96 94 167-269 59-153 | 0 |
| SMcr8 | 97 73 74-147 138-211 |  | 89 102 7-135 8-113 |
| NPRL2 | 0 | 0 |  |
| DENN | 14 93 143-276 224-316 | 79 55 127-181 7 - 62 | 100 274 3-359 71-365 |
| C9orf72 | 60 178 193-398 22-210 | 26 40 195-235 717-756 | 86 134 8-166 17-162 . |
| FNIP (*Ce*) | 72 196 39-286 9-213 | 0 | 0 |
|  |  |  |  |
|  | TARGET | | |
|  | DENN | C9ORF72 | FNIP (*Ce*) |
| QUERY ⇓ | pSS N <query><target> | pSS N <query><target> | pSS N <query><target> |
| FLCN | 60 71 347-424 169-252 | 3 53 364-416 224-277 | 7 84 481-569 862-953 |
| SMcr8 | 26 100 713-821 174-278 | 76 175 721-911 221-457 | 0 |
| NPRL2 | 20 349 72-446 4-450 | 91 90 4-99 18-120 | 0 |
| DENN |  | 14 441 5-491 12-589 | 0 |
| C9orf72 | 36 222 37-289 451-719 |  | 91 92 334-433 858-953 . |
| FNIP (*Ce*) | 76 220 130-435 94-322 | 96 223 236-480 216-463 |  |

Table of hits as in A. Matches for FLCN’s solved structure 3v42 are indicated by asterisks. The top hits to members of the DENN family were: SMcr8🡪3tw8 (DENND1B), NPRL2🡪DENND1C, FNIP🡪DENND1A.

Specific variations in addition to those described in A: for SMcr8🡪FLCN the worm sequence was used as query; for C9orf72🡪SMcr8, NPRL2 and FNIP, the query list was the same 29 sequences used for LCHN and AVL9 (see part A); for C9orf72🡪FNIP the FNIP was human; for FNIP🡪FLCN the query was 300 aa from the C-terminus of T04C4.1a with gaps, consisting of 465-534 / 587-618 / 758-955; for NPRL1🡪C9ORF72 the query was Npr2p (budding yeast).

**Supplementary Table S3: Summary of pairwise alignments of profiles in HHalign**

Name (n) range Prob(%) E-value Cols range (n) Name

**FNIP** (505) 48-505 98.7 6E-11 370 37-465 (481) **C9ORF72**

**FNIP** (505) 2-305 97.6 2E-07 217 9-267 (492) **Lst4p**

**FLCN** (507) 67-139 95.0 4E-07 73 121-194 (787) **SMcr8**

Alignments based on each of the sequences were made in HHblits (8 iterations) and compared in HHalign using default settings. The profile of FNIP (*C. elegans*, T04C4.1a) was aligned to either the profile of Lst4p or the profile of C9ORF72. For FNIP and Lst4p inserts were excised from Lst4p, as defined elsewhere (Supplementary Figure 4C). For the aligned sequences, the total number of residues submitted (n), and the range of residues aligned are shown. In each alignment, the p^SS^ value (Prob %) and the statistical significance of the match (E-value) is shown together with the number of columns (Cols) matched. The strong link between FNIP and Lst4p is mirrored by high p^SS^ values in HHpred of 98% in both directions.

**Supplementary Table 4: Protein sequences used in optimised C9ORF72 alignment**

UNIPROT

REF (species)

Q95QK5 (Caenorhabditis­ elegans)

F1KWH6 (Ascaris suum)

E1G3Y4 (Loa loa)

A8Q582 (Brugia malayi )

E5SEQ0 (Trichinella spiralis )

A7SJN8 (Nematostella vectensis )

H2RQP6 (Takifugu rubripes)

F6S7Y3 ( Xenopus tropicalis)

H2RQP7 (Takifugu rubripes)

F1KWH6 (Ascaris suum)

B3S568 (Trichoplax adhaerens )

E9CDW7 (Capsaspora owczarzaki )

F4PLK8 (Dictyostelium fasciculatum )

F2UBD7 ( Salpingoeca sp)

Q55EM9 (Dictyostelium discoideum )

D2VZQ9 (Naegleria gruberi )

F0ZQQ2 (Dictyostelium purpureum )

D3BNU8 (Polysphondylium pallidum)

E9H292 (Daphnia pulex)

A2EE37 (Trichomonas vaginalis )

A2F138 (Trichomonas vaginalis )

A2DCT2 (Trichomonas vaginalis)

A2EZ61 (Trichomonas vaginalis )

A2E605 (Trichomonas vaginalis )

A2FH28 (Trichomonas vaginalis )

A2DFH5 (Trichomonas vaginalis)

A2E7X1 (Trichomonas vaginalis )

A2DHE9 (Trichomonas vaginalis)

A2DPM1 (Trichomonas vaginalis)

**Supplementary References**

Chou, K.C. (2000). Prediction of tight turns and their types in proteins. Anal Biochem *286*, 1-16.

Kallberg, M., Wang, H., Wang, S., Peng, J., Wang, Z., Lu, H., and Xu, J. (2012). Template-based protein structure modeling using the RaptorX web server. Nature protocols *7*, 1511-1522.

Karplus, K. (2009). SAM-T08, HMM-based protein structure prediction. Nucleic Acids Res *37*, W492-497.

Kelley, L.A., and Sternberg, M.J. (2009). Protein structure prediction on the Web: a case study using the Phyre server. Nature protocols *4*, 363-371.

Ko, J., Park, H., Heo, L., and Seok, C. (2012). GalaxyWEB server for protein structure prediction and refinement. Nucleic Acids Res *40*, W294-297.

Livingstone, C.D., and Barton, G.J. (1993). Protein sequence alignments: a strategy for the hierarchical analysis of residue conservation. Computer applications in the biosciences : CABIOS *9*, 745-756.

Makarenkov, V. (2001). T-REX: reconstructing and visualizing phylogenetic trees and reticulation networks. Bioinformatics *17*, 664-668.

Nookala, R.K., Langemeyer, L., Pacitto, A., Ochoa-Montano, B., Donaldson, J.C., Blaszczyk, B.K., Chirgadze, D.Y., Barr, F.A., Bazan, J.F., and Blundell, T.L. (2012). Crystal structure of folliculin reveals a hidDENN function in genetically inherited renal cancer. Open Biol *2*, 120071.

Roy, A., Kucukural, A., and Zhang, Y. (2010). I-TASSER: a unified platform for automated protein structure and function prediction. Nature protocols *5*, 725-738.

Wu, X., Bradley, M.J., Cai, Y., Kummel, D., De La Cruz, E.M., Barr, F.A., and Reinisch, K.M. (2011). Insights regarding guanine nucleotide exchange from the structure of a DENN-domain protein complexed with its Rab GTPase substrate. Proc Natl Acad Sci U S A *108*, 18672-18677.

Xu, D., and Zhang, Y. (2012). Ab initio protein structure assembly using continuous structure fragments and optimized knowledge-based force field. Proteins *80*, 1715-1735.
